# Pregnancy-related Deaths in Rural Rajasthan, India: Exploring Causes, Context, and Care-seeking Through Verbal Autopsy

**DOI:** 10.3329/jhpn.v27i2.3370

**Published:** 2009-04

**Authors:** Kirti Iyengar, Sharad D. Iyengar, Virendra Suhalka, Kalpana Dashora

**Affiliations:** Action Research and Training for Health, 772 Fatehpura, Udaipur 313 004, Rajasthan, India

**Keywords:** Behaviour, Care-seeking, Maternal mortality, Pregnancy, Pregnancy complications, Rural health, Verbal autopsy, India

## Abstract

In 2002-2003, all deaths (n=156) of women aged 15-49 years in a block of southern Rajasthan were investigated to determine the cause of death and care-seeking behaviour. Family members of 156 (98%) of 160 deceased women were interviewed following the comprehensive listing of all deaths among women of reproductive age. Of the 156 deaths, 31 (20%) were pregnancy-related; 77% of these women died during the postpartum period, and 74% of the deaths occurred in the home. Direct and indirect obstetric causes were responsible for 58% and 29% of the deaths respectively; 12% were injury-related deaths. Medical care was sought for 65% of the women, and 29% were hospitalized. Family perception of not being able to afford treatment at distant hospitals was a major barrier to seeking care, and 60% of those who sought care had to borrow money for treatment. Lack of skilled attendance and immediate postpartum care were major factors contributing to deaths. Improved access to emergency obstetric care facilities in rural areas and steps to eliminate costs at public hospitals would be crucial to prevent pregnancy-related deaths.

## INTRODUCTION

Rajasthan, India's largest state in India in terms of land area, and home to about 6% of its population, has a maternal mortality ratio (MMR) of 445 per 100,000 livebirths, which is substantially higher than the national figure of 301 (1). Since the large majority of maternal deaths occur outside hospitals, there is paucity of information on the causes and circumstances surrounding them. This gap could be addressed by using verbal autopsy, a technique in which relatives of the deceased person are interviewed regarding the conditions and care-seeking sequence preceding death. This information is used to reconstruct the course of illness and for assigning a probable cause of death (2). The technique has been widely used for ascertaining the cause of death in settings in which registration of deaths is low (3-5). Verbal autopsy can additionally provide rich information on how care was sought before death. For assessing maternal deaths, the verbal autopsy method has been found to have high degree of specificity but low sensitivity (6). To facilitate identification of maternal deaths in circumstances in which attribution of cause might be inadequate, ICD-10 introduced the category of ‘pregnancy-related death', defined as the death of a woman while pregnant or within 42 days of termination of pregnancy, irrespective of the cause of death. Hence, in practical terms, there are two approaches to identifying maternal deaths—one based on the medical cause following the ICD definition of maternal death and the other based on timing of death relative to pregnancy, using the ICD-10 definition of pregnancy-related death.

Action Research and Training for Health (ARTH), a non-profit public-health organization based in southern Rajasthan, carried out a verbal autopsy study of all deaths among women of reproductive age (WRA) (15-49 years) residing in one rural block (population 142,379) over a one-year period (7). The objectives were to identify the major causes of death among rural women (aged 15-49 years) in southern Rajasthan and to describe care-seeking patterns, especially the influence of social, economic and gender factors on care-seeking. We classified deaths of WRA in three categories: pregnancy-related deaths, illness-related deaths, and injury-related deaths. The two latter categories excluded women who were pregnant and women whose pregnancy had terminated up to 42 days before death. In this paper, we focus on pregnancy-related deaths.

## MATERIALS AND METHODS

The southern Rajasthan region comprises five of the 32 districts of the state, with a combined population of 8,033,092 residing in 39 blocks. The region has a high proportion of inhabitants belonging to marginalized socioeconomic groups (scheduled castes and tribes) that are characterized by high levels of poverty and illiteracy. Based on selection criteria of 100% rural setting, we narrowed to 14 of the 39 blocks and used female literacy as a marker of status of women. These 14 blocks had an average female literacy rate of 30.2% (range 29.3-31.3%). We purposively selected one block with a female literacy rate of 31.3% for the study. The block had a total population of 142,379, with 37% belonging to marginalized groups (compared to 53% for southern Rajasthan). The study covered deaths that had occurred over a 12-month period from June 2002 to May 2003. Interviews with families were carried out during July-December 2003, up to one year after death.

Five questionnaires were developed and field-tested on a few bereaved families living outside the study area. They included a general information form, one form each for pregnancy-related death, death due to illness, and injury-related death, and a form for care-seeking. Some changes were introduced as a result of field-testing. For example, the care-seeking form which was initially based on institutions was later made provider-based, after we realized that some care providers might visit a patient at home, while some others might treat from their own homes. All forms had translations in the local Mewari dialect in addition to the state language— Hindi. House listers were trained over three days while field investigators received a combination of classroom and practical training on field over seven days.

For listing and enumerating deaths, two house listers visited the main village and every major hamlet of all 160 villages of the study block from where they identified key informants [Auxiliary Nurse Midwives (ANMs), *Anganwadi* (child nutrition) workers, *panchayat* (local self-government) members, shopkeepers, and 2-3 elderly women]. They inquired about all women aged about 12 to 60 years who had died over the past one year and narrowed down to deaths among women aged 15- 49 years. Preparation of the list took about three months from June to August 2003.

For validating lists and conducting interviews with families, a team of two research investigators (with postgraduate degree in social sciences—one female and one male) and one research supervisor (male; with postgraduate degree in social sciences) visited each village and contacted the families of listed deceased women, and verified the date of death and her age. They also inquired whether there had been any other deaths among women in the village during the study period and accordingly revised the list. The investigators made up to three visits in an effort to meet reliable respondent(s) who had been with the woman during the terminal events leading to death.

Verbal consent was taken from one or more elder family members and the respondent to conduct interviews. The investigators offered to return at a convenient time in case the respondent was unable or unwilling at the time of visit. All information relating to identity of women, care providers, and treatment facilities was concealed or changed in the report. The study design was reviewed and approved by the Institutional Ethics Committee of the Action Research and Training for Health (ARTH). The general information form enabled the research investigator to determine whether the death was from illness or injury or was pregnancy-related. In case the investigator felt that the death was attributable to more than one category, forms for both were filled up, and a doctor later decided which one to retain. Every week, the research supervisor reviewed the filled-up forms. If additional information was needed, the team made additional visits to the house or to wherever the respondent was located at the time. For a few women who had been admitted to or had died in hospitals, we tried to get information from the hospital staff on the cause of death. However, the staff was often reluctant to share such information.

Initially, one doctor from the ARTH (the first author) assigned the cause of death. Each form was then sent to one external reviewer from a panel of reviewers, and the cause assigned by the first doctor was hidden (blinded). For pregnancy-related deaths, the external reviewer was a gynaecologist while, for other deaths, the external reviewers included one surgeon and two physicians. If there was disagreement in the opinion of the internal and external reviewers, it was resolved by discussions between the two. The data were entered and analyzed using the Epi Info software (version 6.04).

## RESULTS

At the time of the interview, the investigators detected three deaths that had been missed during listing. Ultimately, a total of 190 deaths were enumerated. Of all the enumerated deaths, 15 were women aged above 49 years at the time of death, 10 had died outside the one-year reference period, and five had been enumerated twice. Hence, 160 were eligible for inclusion in the study. Of these, three families refused, and one had migrated; hence, 156 (98%) interviews could be carried out. The recall period ranged from 4 to 18 months (mean 11.8 months, median 12 months).

Of the 156 deaths among WRA in our study area, 68% were due to an illness, 12% due to injuries (accidents, suicides, homicides), and 20% due to pregnancy-related causes. The most common cause of death among WRA was tuberculosis (TB), responsible for 26% of all deaths (including 10% of pregnancy-related deaths). Maternal deaths contributed to 17.3% deaths among WRA.

We estimated the number of women in the reproductive age-group to be 34,171—24% of the population (7). Going by this, the death rate among WRA worked out to 4.56 deaths per 1,000 population.

To calculate the MMR, we estimated that 4,837 births occurred over one year based on the birth rate in the district, which was 33.3 per 1,000 (8). We deducted 189 stillbirths from among these [based on the World Health Organization's estimates of 39 stillbirths per 1,000 births in India (9)] leaving us with 4,648 livebirths. Given the finding of 31 pregnancy-related deaths, which included 27 maternal deaths in the same year, we estimated the MMR at 581 per 100,000 livebirths for the study block over the previous year. The equivalent ratio in relation to pregnancy-related deaths was 667 per 100,000 livebirths.

### Profile of deceased women

All 31 women who had suffered pregnancy-related deaths were married; one had been widowed during pregnancy (Table 1). Seven women had undergone a second (traditional) marital alliance (*naata*), which implies a failed first marriage. Most deaths had occurred between the age of 20 and 39 years, when most births occur. Although only 37% of the population of the block belonged to the marginalized scheduled castes and scheduled tribes (7), 74% of deaths occurred among women belonging to these groups. The difference between numbers of children born and alive implies high levels of child mortality. The large majority (77%) lived in houses without masonry construction, 6% of families had running water, and only 20% had electricity—all indications of poor socioeconomic status. More than a fourth of the husbands lived and worked in another city. At the time of interview, only 14 (45%) deaths had been registered by the government civil registration system.

**Table 1.** Profile of women who had pregnancy-related deaths (n=31)

Background characteristics	No. (%)
Marital status	
Currently married	30
Widowed	1
Customary remarriage (*naata*)	7
Age-group (years)	
15-19	1 (3)
20-29	15 (48)
30-39	15 (48)
Median age (range) (years)	29 (18-39∗)
Caste group	
Scheduled castes	5 (16)
Scheduled tribes	18 (58)
Other	8 (26)
Mean number of total births	2.8
Mean number of living children	1.7

∗Age range

### Timing and cause of pregnancy-related deaths

The large majority (n=24, 77%) of women died in the postpartum period; of them, 48% died within seven days of childbirth while another 29% died within 8-42 days after delivery. Of those who died in the postpartum period, five women (16%) died within six hours of childbirth, revealing a very high risk during delivery and within a few hours of birth. Five women died in pregnancy, and two died after an abortion. Fifty-eight percent of all pregnancy-related deaths were due to direct obstetric causes, 29% due to indirect causes, and 13% due to injuries. The single most common cause of death was postpartum haemorrhage, followed by sepsis, TB, and anaemia (Table 2). On comparing the cause of death by timing of death, we found that most deaths due to postpartum haemorrhage occurred within 24 hours of birth. Deaths due to injuries occurred from drowning in a well, burns, and snake-bite. We were unable to ascertain if the drowning or burns were of non-accidental nature. Complications leading to death had commenced during pregnancy, delivery, or within seven days of delivery in all but one instance of puerperal sepsis.

**Table 2.** Cause of pregnancy-related deaths by phase of pregnancy

Cause of death	No. of deaths	Phase of death	Subtotal
Antepartum haemorrhage	1	During pregnancy	5
Anaemia	1		
Malaria	1		
Injury (drowning)	2		
Septic abortion	2	Post-abortal	2
Postpartum haemorrhage	7	Immediate postpartum (within 24 hours of delivery)	7
Postpartum haemorrhage	1	2-7 days after delivery	8
Puerperal sepsis	4		
Anaemia	1		
Injury (snake-bite, burns)	2		
Postpartum haemorrhage	1	8-42 days after delivery	9
Puerperal sepsis	2		
Anaemia	1		
Tuberculosis	3		
Heart disease	1		
Hepatitis	1		

### Use of maternal health services

Eighteen women had received antenatal care—11 (62%) from nurses and seven from doctors (38%). Structured questions revealed deficiencies in the quality of antenatal care: although all women had received tetanus immunization, only four had received an abdominal examination, and two received a combination of blood pressure, blood test (probably haemoglobin estimation), and urine examination.

**Fig. 1.**
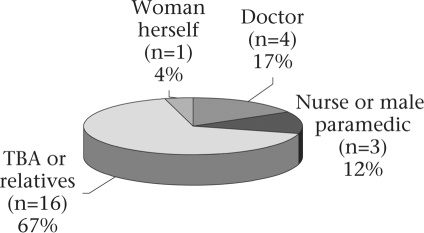
Birth attendants (n=24)

Of the 24 women who had died after childbirth, 19 (79%) had delivered in the home. Birth attendants mostly comprised traditional birth attendants (TBAs) or relatives, and one woman had to manage on her own without any help (Fig. 1). In the two instances when professionally-trained attendants were present during delivery in the home, they left the woman's home some minutes after delivery. These two women developed postpartum haemorrhage and died one hour and three hours after delivery.

TBA=Traditional birth attendant

### Care-seeking for emergencies

Families of 11 women did not seek care from any modern care providers (Table 3). They included four women with injuries, with whom there was little time to seek care, three instances in which the family did not suspect that the situation was life-threatening, and four in which the family perceived that it could not afford the high cost of seeking emergency care.

**Table 3.** Place of pregnancy-related deaths by care-seeking actions (n=31)

Care-seeking action	No. of women	Place of death		
		Home	Health facility	In transit
Did not seek care from any modern providers	11 (35)	11	0	0
Contacted a traditional provider	3	3	0	0
Did not contact any providers	8	8	0	0
Contacted a modern provider	20 (65)	12	5	3
Called provider(s) home	6	5	0	1
Went to a health facility	14	7	5	2
Outpatient treatment only	5	3	1	1
Admitted for inpatient treatment	9	4	4	1

Figures in parentheses indicate percentages

**Fig. 2.**
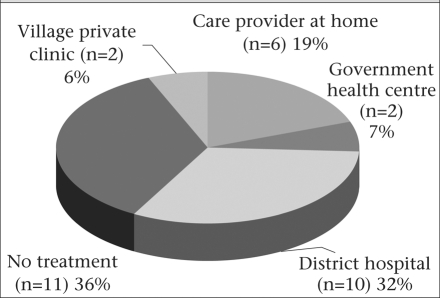
Highest level of treatment sought by women (n=31)

Of the remaining 20, the families of seven invited a modern care provider (nurse or village practitioner) home to treat the woman, and 13 elected to visit a facility. Nine of the latter were taken to a local facility (4 to a village-level clinic, 4 to a government health centre) and five were taken directly to a hospital in the city. The finding that five women received only outpatient treatment for life-threatening conditions points to the inability of the care provider to gauge the seriousness of the condition and/or inability of the family to afford inpatient care. Eventually, 14 women were taken outside home to seek care. Ten of these women did ultimately reach a referral hospital, of whom nine were admitted. The three deaths that occurred in transit (due to postpartum haemorrhage, puerperal sepsis, and abortion) were related to delayed decisions to transport the women. The highest health facility most commonly visited was the district hospital. However, many families limited themselves to calling a provider to home, or to visiting a village private clinic or a rural health centre. Figure 2 highlights the observation that care-seeking for life-threatening conditions among women was very deficient in the study area.

For women who did seek care, the median distance to each facility was 20 km while the mean distance was 33 km. City-based comprehensive emergency obstetric facilities were located at a distance of 45-100 km from various parts of this block.

Care-seeking patterns reflected a combination of influences, including the severity and progression of the complication, availability of family members and their perception of the costs of travel and emergency care, the actual cost of care, the advice of care providers about referral, and the value attached in general to the woman and her pregnancy. The variance and interplay among these factors can be discerned in review of seven individual narratives presented below (all names have been changed).

#### Sonki Daluram

Sonki, a 24-year old woman of a tribal community, lived in a village 54 km away from the district town. Her family had noticed that even before becoming pregnant, she would feel weak and tired while working. She looked pale and had chronic cough. After becoming pregnant for a second time, Sonki received three antenatal care examinations from the local government ANM who also gave her tetanus injections and iron tablets. The family was not aware about whether or not Sonki actually consumed those tablets. In the seventh month of pregnancy, Sonki started getting fever and headache, and a month later, also found it difficult to see in the dark (night blindness). Meanwhile, her cough, weakness, and pallor persisted. At full term, Sonki delivered a baby at home, assisted by a TBA. Soon after delivery, she felt dizzy, was unable to stand, and even fell once on trying. Two days after delivery, her breathing became rapid, and the cough grew severe. Another two days later she developed high fever, with pain and swelling in the legs. Two weeks after she delivered, Sonki's family took her to a government hospital 29 km away where, after chest x-ray and sputum test, the doctor diagnosed TB. Sonki was given an injection and some drugs and was asked to return after three days. The family had borrowed Rs 1,150 (US$ 25) for the tests and treatment. Sonki could not follow up because of a death in the family but continued to worsen. On finding her very weak 10 days later, her family took her to a specialized government TB hospital 90 km away where she was hospitalized and investigated again. The doctor confirmed TB and prescribed some tablets, injections, and an intravenous drip. Three days later, Sonki died in the hospital. Her family had again borrowed Rs 3,000 (US$ 67) to pay for transportation and treatment. Her baby died two months later.

Sonki died from pulmonary TB and anaemia that remained undetected throughout pregnancy, notwithstanding three antenatal examinations. When TB was diagnosed two weeks after delivery, despite belonging to a socioeconomically-marginalized group, she did not receive free treatment at two public facilities covered by the Revised National Tuberculosis Control Programme of India. Two weeks after delivery, the first hospital treated her as an outpatient at a time when inpatient care would probably have been appropriate. Sonki's family ended up spending substantial sums for which they had to borrow at short notice.

#### Champa Kaluram

Champa, a 32-year old woman from a tribal community lived in a village 53 km from the district town. She had three children, all in reasonably good health and was pregnant again. She did not receive any antenatal check-ups or tetanus injections. Once during the eighth month of pregnancy, she went to the hills to graze cattle and collect firewood. Apparently, her husband had advised her not to. Champa collected a large pile of wood and was bringing it downhill when she slipped and fell. She left the wood and came home. She started to have vaginal bleeding soon thereafter but did not inform anyone because she was afraid of her husband's reprimand. She carried on with household work for two days and informed her sister-in-law (*devarani*) and mother-in-law only when the bleeding worsened. There was not enough money for treatment; so, her mother-in-law sent her younger son to call Champa's husband who was working in a town nearby. She also called for a TBA. The TBA unsuccessfully tried to deliver the baby but Champa continued to bleed. Her husband arrived at night and decided to take her to a hospital next morning after arranging for some money. However, Champa died the same night without giving birth. The family believed that the baby had died earlier, and its poison had spread to Champa. According to her mother-in-law and sister-in-law, “If she had told us in time, we could have helped her carry the wood. And then we did not have enough money. After all, how is it possible to get someone treated without money? You need money for treatment.”

Champa's medical cause of death was antepartum haemorrhage. It was, however, evident that lack of cash and a decision-maker prevented her family from immediately accessing transport and emergency treatment. The family carried the impression that emergency services at the government hospital would cost a lot of money—even her husband, who had migrated for work, had planned to borrow money before taking her to a hospital.

#### Yashoda Sohanlal

Yashoda, a 24-year old mother of three children (of whom two had survived), had missed her period. She underwent a urine test to confirm pregnancy at a private clinic. Yashoda came home and decided not to continue the pregnancy. She consulted some neighbours who advised her to contact a certain woman belonging to a nomadic tribe, for medicines that would abort the current pregnancy and prevent future ones. A few days after visiting this woman, Yashoda developed high fever, vomiting, abdominal distension, and swelling of hands and feet. When she started fainting repeatedly, she was taken to her parents' house from where she was rushed to the district hospital (a comprehensive emergency obstetric care facility). The doctor there initiated treatment and recommended that she be shifted to the government teaching hospital, 70 km away. The family spent Rs 950 (US$ 24) at the district hospital. However, instead of taking her to the teaching hospital, the family preferred getting Yashoda admitted to a private hospital nearby where she was given intravenous fluids, oxygen, and a blood transfusion. Yashoda delivered a premature foetus that survived for two hours. After 10 days of inpatient treatment and spending Rs 40,000 (US$ 1,000), the family was advised by the doctor to take her to the government teaching hospital. Although Yashoda was semi-conscious, her intravenous lines were disconnected and she was discharged. The family transported her to the teaching hospital (also a comprehensive emergency obstetric care facility) where she was admitted but the doctor informed them that there was little hope of survival. The family brought Yashoda home where she died after a day.

Yashoda died as a consequence of septic unsafe abortion. She resorted to an unsafe mid-trimester abortion in a block that had only one legal private facility providing first trimester services; the government health centre did not offer any abortion services. The government district hospital (a comprehensive emergency obstetric centre) referred her soon after admission, and the private hospital was unable to stabilize her despite 10 days of treatment.

#### Tamli Nokharam

Tamli, a 27-year old mother of three lived in a subdistrict town. She had visited the local Community Health Centre (CHC) twice for antenatal check-up and had received tetanus injections and iron tablets. She was in good health and carried on with routine work through pregnancy. In the ninth month, she started having some abdominal pain which persisted for three days. Accompanied by her husband and a neighbour, Tamli walked to a primary health centre (PHC), 3 km away to see a doctor they were familiar with. After examining her, the doctor informed them that the foetus was already dead and advised them to go to the teaching hospital, 73 km away. Tamli's husband did not agree, saying that they could not afford to go and pleaded with the doctor to manage her there. The doctor grudgingly agreed, saying that the responsibility for any consequences would not be his. With the help of intravenous (IV) fluids and tablets, the doctor and a nurse induced labour, and Tamli delivered a stillborn baby within two hours. Soon thereafter, she started bleeding profusely. According to a family member, “all her clothes were soaked, the cot was soaked, the mattress was soaked, and the bucket was full” (of fluid and blood). Tamli died two hours after delivery. A neighbour recalled during the interview, “She went walking to the hospital that morning, and a few hours later, they brought her home dead.”

Tamli died from postpartum haemorrhage following induction of labour for intrauterine foetal death, in a PHC. It is possible that, in addition to drugs to induce labour, some manipulation was also carried out.

#### Motki Bhuraram

Twenty-six years old Motki, with no living child from two previous deliveries, was pregnant again. Around the fourth month of pregnancy, she began to look pale and developed frequent headache. In the sixth month, she started having fever, shortness of breath while working, and blurred vision. In the seventh month, Motki developed swelling of face and hands, abdominal pain, and fast breathing and also lost her appetite. Her family recalled that she slept with difficulty in the month before delivery and preferred to remain in the sitting position. In eighth month, Motki had consulted an ANM who suspected jaundice and advised her to go to the district hospital. Six days later, her family took her to a local government PHC where, after a blood test, she received IV fluids. After about 4-5 hours and an expenditure of Rs 900, the family was advised to seek higher-level treatment in the city. However, the family could not afford to take her to the city and instead took her home. Four days later one evening, Motki went into labour. The family called an ANM who, however, refused to come. Motki delivered at night with the assistance of family members but the baby was stillborn. Soon after the placenta was delivered, she started bleeding profusely. Next morning she was taken to the government district hospital where she was given medicines, injections, and oxygen but blood-transfusion facility was not available. The following day at around 4 pm, Motki died in the hospital while on treatment. The family had borrowed to spend about Rs 1,000 (US$ 25) for her treatment. Apparently, family members had presented their ‘below poverty-line' card that entitles patients to free treatment at government facilities but were told that it would not work there.

Motki apparently died from postpartum haemorrhage compounded by anaemia in pregnancy. Her visits to the ANM and PHC during pregnancy apparently did not help detect and manage anaemia. The family did not follow the advice to go to a hospital before delivery, perhaps because of the uncertainty relating to an unfamiliar place and the cost of treatment. Motki's family unsuccessfully tried to call an ANM for the delivery and then delayed going to the district hospital even after severe bleeding started due to a perceived lack of sufficient money to pay for emergency treatment. Lastly, the district hospital was unable to arrange blood transfusion in an emergency

#### Kanni Roop Singh

Twenty-two years old Kanni was pregnant for the first time. Over three antenatal care visits to a doctor and nurse at the CHC, she had received tetanus injections and iron tablets. Kanni, however, had bodyache, weakness, and breathlessness. When labour pains commenced, she was taken to the CHC. The doctor suspected transverse lie and referred her to the teaching hospital, 70 km away, and her family took her there in a jeep. At the teaching hospital, Kanni vaginally delivered a stillborn girl and was discharged after 3-4 days. The family had spent Rs 2,200 at the hospital. Three to four days later at home, she developed fast breathing and malaise. Fifteen days later, she developed swelling of the whole body and lost her appetite. The family took her 30 km away to a reputed unqualified practitioner who advised them to go back to the city hospital. So, her husband and parents-in-law accompanied her to the teaching hospital where some tests were performed. The doctor said that she needed urgent blood transfusion. Kanni's husband was reluctant to donate blood, and his mother flatly refused to let him do so. The doctor then asked Kanni's mother to donate blood but her son (Kanni's brother) refused to let her do so. The doctor even suggested that they could buy blood and cautioned that otherwise she might die. The family discussed the situation and decided that they could not afford it and, hence, brought her home without blood transfusion. Kanni died a day later, 18 days after giving birth. In the words of her mother, “… the girl was frail and had a bent, deformed back (kyphosis) which was not to her (marital) family's liking. Why would her family bother to treat someone they did not like?”

The medical cause of death was severe anaemia with congestive heart failure. Kanni's account underscores the low value accorded to women's lives—her family members were unwilling to donate or arrange blood for her. It is possible that haemoglobin was not tested during pregnancy. It is not clear whether anaemia was recognized and treated at the time of delivery. Lastly, Kanni also did not receive any postnatal care—her condition deteriorated over a fortnight after she delivered.

### Cost of treatment

For pregnancy-related deaths, the mean and median costs incurred per woman were Rs 6,064 (US$ 138) and Rs 1,300 (US$ 30) respectively. Sixty percent of the families had to borrow money to meet expenses. Some families were in debt even by the time of interview as reflected in the following statements:

My wife had swelling onwards from one month before delivery. I was not at home. I received a phone call, so I rushed home. … I sold some land and also borrowed some money. I got her admitted to a private hospital where she delivered after three days. I was in great difficulty around that time. People (creditors) come asking for their money even now, hence I come home after work only late at night. (Husband of a tribal community woman who died on the 5th day after delivery)

I have spent a lot of money—look at these bills. For three months, we were in the hospital, because of which our living conditions have suffered. Even you can see for yourself. (A manual labourer, whose wife died 32 days after delivery)

Some women could not be taken to a hospital for treatment because their families could not afford it, as exemplified by the following statement by the sister-in-law of a woman who died a month after delivery due to anaemia and sepsis:

Her husband did not have money. He thought that he would mortgage his farm and her jewellery and then take her for treatment. But who would take the farm at such a short notice? And there was no one from her parents' family to help. Hence, we could not get her treated.

## DISCUSSION

In the study area in southern Rajasthan, the most common cause of death was TB, which accounted for 26% of all deaths and contributed to 10% of pregnancy-related deaths. World Health Organization has identified TB as the single biggest killer of women aged 15-44 years in the world, accounting for 9% of deaths in this group worldwide (10). Maternal deaths followed, accounting for 17.3% of the total. Research has shown that, in countries with poor health services, maternal deaths account for a higher proportion of all deaths among WRA while, in developed countries, they contribute to less than 1% (11). Studies in Egypt and Indonesia have reported that complications of pregnancy and childbirth caused a large proportion of deaths (12). In Egypt, the most common cause of deaths among WRA was circulatory system disease (28%), followed by complications of pregnancy and childbirth (23%) and trauma (14%), primarily burns. In Indonesia, complications of pregnancy and childbirth were the most common cause of deaths (23%), followed by infectious diseases (22%, primarily TB), and circulatory system disease (13%).

Women of the marginalized groups, such as scheduled castes and scheduled tribes, faced a disproportionately-higher risk of maternal death compared to the better-off sections of the population, primarily because of their inability to access timely treatment. On the other hand, the wide difference between the mean number of children ever-born and living reflects on poor child survival and is a likely contributor to higher, rebound fertility and commensurate greater risk of maternal death.

Twenty-four (77%) pregnancy-related deaths occurred during the postpartum period, including eight within 24 hours. This draws attention to the importance of monitoring during delivery and during the 24 hours immediately thereafter. Delivery by a skilled attendant could logically have helped prevent some of these deaths through better monitoring. The findings of a demographic and health survey revealed that, in 2005-2006, only 43% of women in Rajasthan accessed the services of a skilled attendant (13). We observed that even when professionally-trained providers were present for deliveries in the home, they left within minutes of delivery. The women in question had developed a complication soon and died within 1-3 hour(s). This suggests that, if a skilled attendant were to conduct a delivery in the home, s/he should be available to monitor the woman for at least the first twenty four hours.

Nine women (29%) died between 8 and 42 days after delivery from anaemia and/or sepsis that emerged as complications within the first week. In all but one woman, the complications had commenced within seven days and, hence, were amenable to detection through postnatal examinations during the first week. Careful pre-discharge assessment, including haemoglobin test, followed by 1-2 routine postnatal home-visit(s) could be expected to detect most complications in an early, retrievable stage. The Government of India guidelines (2005) have already recommended two postnatal visits—2-3 days and 8-10 days—after delivery (14). However, this would be possible only if the prevailing low penetration of postnatal care improves substantially (according to the National Family Health Survey 3, 21.7% of women in rural Rajasthan received postnatal care within two days after delivery).

Nearly one-third of pregnancy-related deaths were attributable to indirect obstetric causes. This finding is consistent with the findings of other studies in South Asia (15), which have found that indirect causes were responsible for up to 47% of maternal deaths. Anaemia—highly prevalent in India—contributes to nearly 20% of maternal deaths (8). Several women, who died of anaemia, TB, and heart disease, suffered these conditions during pregnancy but their problem probably remained undetected during antenatal examination. Antenatal care delivered through primary healthcare workers in India is currently guided by a ‘mother-child protection card' that records only tetanus immunization and iron-folate supplementation services for pregnant women. In line with this, antenatal examination remains inadequate and incomplete. We, therefore, recommend that the Government of India's mother-child protection card be revised to include antenatal and postnatal screening of conditions, such as anaemia and TB, and that it further be used for monitoring the quality of antenatal and postnatal care by the health system on a routine basis.

Women found to have TB tried to obtain treatment but high costs proved to be a barrier. Some of them were asked to pay for treatment at a government facility while the rest could not afford the high cost of treatment at private facilities. Better linkages between the national programmes for reproductive and child health (RCH programme) and that for TB (Revised National Tuberculosis Control Programme), and better governance to curb demand for irregular payments would be necessary for more effective control of TB during the maternal period.

About two-thirds of deceased women sought ‘modern' medical care. Most visited care providers at a median distance of 20 km from their homes, using their own transport facilities and paying for them directly. For half of the women with pregnancy-related deaths, the highest level of facility visited neither provided basic emergency services, nor did it proactively arrange for and bear the cost of referral. The programme managers and care providers have often assumed that families will readily take women with complications to distant urban facilities, which are often located 50-60 km away from the village. Our findings suggest that, in the absence of transport arrangements and confidence about the costs being affordable, rural families are unlikely to undertake the trip to a referral hospital. Apart from transport, locating EmOC in rural health centres could, therefore, help avert several maternal deaths.

Sixty percent of the families had to borrow money to meet healthcare expenses. The process of borrowing appears to have contributed to significant delays in treatment. In the extreme situation, families avoided seeking any treatment due to lack of money. Given that services in the public sector are meant to be free, it is essential that action is taken to completely minimize, if not eliminate hidden costs at government hospitals, if access to services for poor rural families is to be increased.

Three-fourths of the pregnancy-related deaths in our study occurred at home. An alarming finding was that several women who did try to seek care outside home eventually died at home. Analysis of these situations showed that some of these women were provided only outpatient treatment, some were discharged prematurely from hospital, and others were brought home against medical advice because their families could not afford further treatment. Family perception of not being able to afford treatment at distant hospitals was a common theme running through our study. This perception is likely to have emerged from the collective past experience of this community, in seeking emergency care from government hospitals. Policy-makers would, therefore, have to grapple with measures not only to reduce the real cost of care-seeking but to also project emergency health services as being genuinely affordable. Such an intervention might require studies of user-costs.

Deaths due to injuries contributed to 13% of pregnancy-related deaths. In western societies, some studies have suggested that intentional injuries and, possibly, unintentional injuries may be more common among women during pregnancy (16). In the UK, suicide is a leading cause of maternal death (17), although suicide rates appear to be much lower during pregnancy and in the first year after childbirth than in women without a recent pregnancy. Some authors have even supported the idea that women may be more prone to injuries during pregnancy (18).

At the district level in settings with high levels of maternal death, verbal autopsy by district administration or health department could be an important step towards initiating corrective measures to improve maternal health services. However, the biggest challenge would be identification of deaths. This study found that only 45% of deaths had been recorded by the civil registration system, at the time of interview.

To summarize, the findings of this study indicate that several factors had contributed to maternal mortality in Rajasthan. Although the high prevalence of health conditions and diseases, including TB and anaemia, are identifiable as direct or indirect causes of death, important societal and health systems factors constrain women from accessing quality health services. If reduction in maternal mortality is to become a reality, women in rural Rajasthan and similar regions will require more efficient access to high-quality delivery and emergency services at an affordable cost.

## ACKNOWLEDGEMENTS

The study has been financially supported by the Mac Arthur Foundation. The funder has had no involvement in the research, writing, or in the decision to submit the paper for publication.
